# Cytokine Responses to Acute Exercise in Healthy Older Adults: The Effect of Cardiorespiratory Fitness

**DOI:** 10.3389/fphys.2018.00203

**Published:** 2018-03-15

**Authors:** Mark T. Windsor, Tom G. Bailey, Maria Perissiou, Lara Meital, Jonathan Golledge, Fraser D. Russell, Christopher D. Askew

**Affiliations:** ^1^VasoActive Research Group, School of Health and Sport Sciences, University of the Sunshine Coast, Sunshine Coast, QLD, Australia; ^2^Centre for Research on Exercise, Physical Activity and Health, School of Human Movement and Nutrition Sciences, The University of Queensland, Brisbane, QLD, Australia; ^3^Queensland Research Centre for Peripheral Vascular Disease, James Cook University, Townsville, QLD, Australia; ^4^Department of Vascular and Endovascular Surgery, Townsville Hospital, Townsville, QLD, Australia

**Keywords:** inflammation, interleukin-6, interleukin-10, tumor necrosis factor-α, aging

## Abstract

Markers of chronic inflammation increase with aging, and are associated with cardiovascular disease prevalence and mortality. Increases in fitness with exercise training have been associated with lower circulating concentrations of cytokines known to have pro-inflammatory actions (such as interleukin-6 [IL-6]) and higher circulating concentrations of anti-inflammatory cytokines (interleukin-10 [IL-10]). However, the effect of cardiorespiratory fitness on acute cytokine responses to a single bout of exercise in healthy older individuals is unknown. We compared the response of plasma cytokines IL-6, tumor necrosis factor-alpha (TNF-α) and IL-10 to a bout of moderate-intensity continuous and higher-intensity interval exercise between older individuals with higher and lower levels of cardiorespiratory fitness. Sixteen lower-fit (*V*O_2peak_: 22.6±2.8 mL.kg^−1^.min^−1^) and fourteen higher-fit participants (*V*O_2peak_: 37.4±5.9 mL.kg^−1^.min^−1^) completed three 24 min experimental protocols in a randomized order: (1) moderate-intensity continuous exercise (40% of peak power output [PPO]); (2) higher-intensity interval exercise (12 × 1 min intervals at 70% PPO separated by 1 min periods at 10% PPO); or (3) non-exercise control. Plasma cytokines were measured at rest, immediately after, and during 90 min of recovery following exercise or control. Plasma IL-6 concentrations at baseline were greater in the higher-fit compared to the lower-fit group (*P* = 0.02), with no difference in plasma IL-10 or TNF-α concentrations at baseline between groups. Plasma IL-6 and IL-10 concentrations in both groups increased immediately after all protocols (IL-6: *P* = 0.02, IL-10: *P* < 0.01). However, there was no difference in the IL-6 and IL-10 response between the exercise and non-exercise (control) protocols. After all protocols, no changes in plasma TNF-α concentrations were observed in either the higher- or lower-fit groups. In this study, basal concentrations of circulating IL-6 were elevated in older individuals with higher levels of cardiorespiratory fitness. However, changes in plasma cytokine concentrations after exercise were not different to changes after non-exercise control in both the lower- and higher-fit groups.

## Introduction

Chronic, low-grade inflammation is a common feature of various age-related chronic diseases (Himmerich et al., 2006). Such diseases are a major cause of global morbidity and mortality (Das et al., 2017), and this burden is expected to increase with an aging population (Yazdanyar and Newman, 2009). Regular exercise facilitates the creation of an anti-inflammatory environment (Petersen and Pedersen, 2005), leading to reduced basal inflammatory-, and increased anti-inflammatory, cytokine concentrations in both younger and older adults (Monzillo et al., 2003; Goldhammer et al., 2005; Santos et al., 2012). These long-term changes in circulating cytokines are believed to be mediated by the repeated acute inflammatory, and subsequent anti-inflammatory cytokine responses during recovery from single bouts of exercise (Reihmane and Dela, 2014).

In response to a short bout of exercise, acute increases in IL-6 have been proposed to suppress pro-inflammatory TNF-α (Starkie et al., 2003), and up-regulate anti-inflammatory cytokines such as IL-10 (Steensberg et al., 2003; Lira et al., 2009), creating an anti-inflammatory milieu for several hours after the cessation of exercise (Mendham et al., 2015). While this response is well established in young people, the response in older adults is more varied, with reports that the skeletal muscle (Hamada et al., 2005) and circulating (Reihmane et al., 2013, 2016) cytokine responses to a single bout of exercise may be supressed compared with those observed in young participants.

Unlike the anti-inflammatory benefits of transient increases in IL-6 from skeletal muscle with acute exercise, elevations in IL-6 released from other sources (e.g., hepatocytes or adipose tissue), are associated with increases in pro-inflammatory TNF-α and C-reactive protein (CRP). Importantly, higher levels of cardiorespiratory fitness have been associated with lower circulating concentrations of both IL-6 and CRP at rest (Kohut et al., 2006), which raises the possibility that low cardiorespiratory fitness may also contribute to the cytokine response to acute exercise in older adults. In younger adults, who typically have higher levels of cardiorespiratory fitness, the cytokine response to exercise does not appear to be influenced by cardiorespiratory fitness (Scott et al., 2013; Landers-Ramos et al., 2014). However, few studies in middle-aged or older adults have attempted to directly address this question. In recreational cyclists (age: 43 ± 10 y) higher self-reported fitness was associated with a lower CRP response to prolonged competitive exercise (Kleiven et al., 2017). In contrast, a recent study comparing masters athletes with lower-fit untrained adults reported no difference in the anti-inflammatory IL-10 and TGF-β responses to a single bout of exercise (Minuzzi et al., 2017). Whether cardiorespiratory fitness influences the acute inflammatory response to exercise in older adults remains to be established.

Interval exercise enables periods of higher-intensity exercise interspersed with periods of recovery, beyond the intensity that could normally be sustained with continuous exercise. This format of exercise is increasingly being used and recommend as part of the prevention and management of various chronic conditions (Gibala et al., 2012). In older adults, high-intensity interval training has been reported to elicit greater improvements in cardiorespiratory fitness compared with continuous exercise (Hwang et al., 2016); and in young adults interval exercise training is reported to exert similar improvements in cardiometabolic health, despite a lower overall exercise volume (Gillen et al., 2016). Acute increases in IL-6 were reported to be augmented in young adults after high-intensity interval exercise compared to moderate-intensity continuous exercise of the same duration and workload (Leggate et al., 2010). In contrast, a recent study of older adults suggests that the IL-6 response to interval exercise was lower than that during work- and time-matched continuous exercise (Windsor et al., 2017), although this study only included individuals with relatively low levels of cardiorespiratory fitness.

We hypothesize that low levels of cardiorespiratory fitness may contribute to the suppressed inflammatory responses to exercise previously reported in older adults. Understanding the interaction between cardiorespiratory fitness, exercise intensity and the cytokine responses to short-term exercise in older adults may improve exercise training prescription to optimize the anti-inflammatory effects of exercise as part of the prevention and management of age- and inflammatory-related conditions. Therefore, the aim of this study was to compare the plasma IL-6, TNF-α, and IL-10 responses to moderate-intensity continuous and higher-intensity interval exercise between healthy older adults with lower and higher levels of cardiorespiratory fitness.

## Methods

### Subjects

Sixteen lower-fit participants and 14 higher-fit participants were recruited from a University alumni cohort and through local advertisement. Participants were included if they were aged 60–86 years, non-smokers (>12 months no smoking history) and able to undertake cycling exercise. Participants were excluded if they had a body mass index (BMI) >39, uncontrolled hypertension (systolic blood pressure ≥140 mmHg and/or diastolic blood pressure ≥90 mmHg), unstable angina or diagnosed reversible cardiac ischemia, diagnosed uncontrolled cardiac arrhythmia with recurrent episodes or symptoms on exertion, heart failure, symptomatic aortic stenosis, or chronic obstructive pulmonary disease. Participants were also excluded if they had a known inflammatory condition including rheumatoid arthritis, ankylosing spondylitis or chronic active hepatitis, or were prescribed anti-inflammatory medication (e.g., NSAIDS) for regular use. Participants were informed of the methods and study design verbally and in writing before providing written informed consent. The study was approved by the University of the Sunshine Coast Human Research Ethics Committee, and conducted in accordance with the *Declaration of Helsinki*.

### Experimental design and study overview

Participants attended a baseline assessment followed by three experimental visits. The baseline assessment involved anthropometric measurements and a maximal incremental cycling test for the determination of maximal work output and cardiorespiratory fitness (*V*O_2peak_). *V*O_2peak_ data were used to stratify participants into either the higher-fit (*V*O_2peak_ ≥ 32 mL.kg^−1^.min^−1^ for males, ≥28 mL.kg^−1^.min^−1^ for females) or lower-fit group (*V*O_2peak_ ≤ 28.0 mL.kg^−1^.min^−1^ for males, ≤ 25 mL.kg^−1^.min^−1^ for females) based upon current normative values for older individuals (Heyward and Gibson, 2014). All participants then attended three additional experimental visits conducted in a randomized, cross-over design. During these visits, participants completed a non-exercise control, a moderate-intensity continuous or higher-intensity interval cycling exercise protocol. All exercise was performed in an upright position on an electro-magnetically braked cycle ergometer (Lode Corival, Lode B.V., Groningen, Netherlands) and each visit was separated by 3–10 days, during which time participants were asked to maintain their habitual diet and physical activity patterns. Participants were asked to refrain from drinking alcohol or consuming caffeine for 12 h, and from using pro re nata anti-inflammatory medication for 72 h prior to each visit. Participants were required to be fasted for 3 h prior to each visit after the consumption of a standardized snack (4 oat breakfast biscuits, 20 g CHO, 8 g fat). Testing was performed at the same time of day under consistent laboratory conditions (room temperature: 23 ± 1°C).

#### Maximal incremental cycling test

Participants cycled for 3 min with no resistance at a self-selected pedal-rate between 60 and 90 RPM, which was then maintained throughout the test. Resistance then increased to 20 W for 1 min, and by a further 10 W each min until volitional cessation. All participants reached the criteria for maximum effort, defined by achieving the following end-points: (1) heart rate within 10 b·min^−1^ of age-predicted peak heart rate, (2) respiratory exchange ratio of >1.15, and (3) rating of perceived exertion (RPE) ≥9 on a 10 point scale (Borg, 1982). Heart rate was recorded continuously using a 12-lead ECG (Mortara Inc., Milwaukee, WI, USA) and RPE was recorded during the final 10 s of each stage. Gas exchange data were collected continuously and stored at 15 s intervals using a Parvo Medics TrueOne 2400 metabolic cart and software (Parvo Medics, East Sandy UT, USA). *V*O_2peak_ was determined as the highest *V*O_2_ value in a given 15 s period over the final minute of exercise. Peak power output was then used to establish the exercise intensity for the subsequent experimental test visits.

#### Experimental testing visits

Each of the three experimental visits (visits 2–4) commenced with the participant lying in the supine position for 15 min. During this time, a 12-lead ECG was fitted, an antecubital forearm vein was cannulated and a resting blood sample was drawn. Participants then completed one of the following protocols: (1) control, rest in an upright seated position; (2) moderate-intensity continuous cycling exercise at 40% peak power output (W); (3) higher-intensity interval cycling exercise, consisting of 12 × 1 min intervals at 70% peak power output separated by 1 min periods of active recovery at 10% peak power output. Participants cycled at a self-selected pedal rate of 60–90 RPM, which was kept consistent between visits. Each protocol was matched for time (24 min), and the exercise protocols were matched for total work. Heart rate and RPE were recorded during the final 10 s of each bout of the interval exercise protocol, and at the same corresponding time (i.e., every 2 min) during the other protocols. After completing the protocol, participants were immediately returned to the supine position and were monitored for 90 min. In order to establish the cytokine responses to each experimental session, blood samples were drawn before and at 3 time points following exercise or control (immediately, 20 and 90 min after).

#### Physical activity recall

Upon arrival at the laboratory for each experimental visit, participants completed the International Physical Activity Questionnaire modified for the elderly (IPAQ-E). Briefly, participants were asked to recall their physical activity levels during the previous 7 days, including the amount of time doing moderate or vigorous physical activity. Examples of the types of physical activity deemed moderate or vigorous were supplied within the questionnaire (Hurtig-Wennlöf et al., 2010).

#### Blood sampling and biochemical analyses

After discarding the first 4 ml of collected blood, samples were collected in K_2_EDTA Vacutainer™ tubes using standard aseptic techniques. Normal saline (0.9%) was used to flush the cannula after each blood draw to maintain patency. Plasma was separated by centrifugation (1,500 × g for 15 min at 22°C) and stored in 1.5 mL aliquots at −80°C until further analysis. For IL-6 and IL-10, an additional time point was included (20 min post-exercise) to ensure that any peak in cytokine concentrations, which is reported to occur shortly after exercise cessation, was captured in the dataset (Petersen and Pedersen, 2006; Gleeson et al., 2011). Plasma IL-6, IL-10, and TNF-α concentrations were measured using commercially available sandwich ELISA kits (eBioscience, San Diego CA, USA). The catalog number for each ELISA kit are as follows; IL-6: 88-7066, IL-10: 88-7106, TNF-α: 88-7346. Samples were analyzed in duplicate for all time points, and all techniques and materials were used according to the manufacturer's instructions. All samples for any one participant were analyzed using the same assay to eliminate inter-assay variance. The within assay coefficient of variation for the duplicate analyses performed were (mean ± *SD*): IL-6 4.7 ± 5.0%; IL-10 4.6 ± 5.3%; TNF 4.3 ± 4.3%. The between visit intra-class correlations for each cytokine at baseline were as follows: IL-6: 0.86 (95% confidence interval 0.75–0.93), IL-10: 0.97 (0.94–0.98), TNF-α: 0.99 (0.97–0.99). The assay sensitivity for each cytokine was calculated by multiplying the control value on each assay by two (IL-6: 2.0 pg/ml, IL-10: 1.4 pg/ml, TNF-α: 6.3 pg/ml).

### Data analysis

Sample size estimates were calculated (G^*^Power software, v 3.1) based on the mean change in IL-6 concentrations in response to short-duration exercise (1.5 ± 0.2 pg/ml) in both older and younger populations (Kinugawa et al., 2003; Castellano et al., 2008; Landers-Ramos et al., 2014). With 80% power and an alpha level of 0.05, an estimated sample size of five subjects per group is needed to detect this same difference between groups and conditions. Comparisons of baseline cytokines between groups were carried out using a two-way linear mixed model (LMM) analysis. Heart rate and RPE during exercise, and cytokine concentrations after each experimental protocol were compared between fitness groups (higher, lower), between protocols (moderate-intensity continuous, higher-intensity interval, control), and across time (before each protocol, immediately post-, 20 min post- and 90 min post-protocol) using a three-way LMM (group^*^protocol^*^time). Data that were not normally distributed were transformed as appropriate before statistical analyses. Statistically significant interactions were further investigated with multiple comparisons using Fisher's least significant difference approach (Rothman, 1990; Perneger, 1998). The strength of the relationships between self-reported physical activity levels, cardiorespiratory fitness levels, cytokine concentrations at baseline and changes in cytokine concentrations (delta) in response to exercise were assessed using Pearson correlation coefficient. Analyses were conducted using the Statistical Package for Social Sciences (Version 22; IBM SPSS Inc., Chicago, IL). Statistical significance was delimited at *P* < 0.05 and exact *P*-values are cited. Data are presented in the text as mean (95% confidence interval) unless otherwise stated.

## Results

### Baseline participant characteristics

The baseline participant characteristics are presented in Table 1. Absolute and relative *V*O_2peak_ were greater in the higher-fit group (*P* < 0.001). There were no significant differences between groups in terms of age, height, body mass and BMI. The higher-fit group reported completing 390 min (95% CI, 105–675, *P* = 0.008) more activity minutes per week than the lower-fit group.

**Table 1:** Participant characteristics and cardiorespiratory fitness.

	**Lower-fit group**	**Higher-fit group**	***P*-value**
**Participants** (*n*)	16	14	–
Male *n* (%)	14 (87.5)	12 (86)	0.65
Female *n* (%)	2 (12.5)	2 (14)	0.65
**ANTHROPOMETRIC**			
Age (years)	72 (6)	69 (5)	0.16
Height (cm)	174 (8)	177 (8)	0.44
Body mass (kg)	77 (13)	77 (7)	0.54
BMI (kg/m^2^)	25 (4)	25 (3)	0.63
**MEDICATION USE**, ***n*** **(%)**			
Calcium channel blockers	3 (19)	3 (21)	0.99
ACE inhibitors	0 (0)	2 (14)	0.21
Statins	5 (31)	2 (14)	0.40
Beta blockers	2 (13)	0 (0)	0.49
**FITNESS/ACTIVITY**			
Absolute *V*O_2peak_ (L.min^−1^)	1.7 (0.4)	2.9 (0.6)	**<0.001**
Relative *V*O_2peak_ (mL.kg^−1^.min^−1^)	22.6 (2.8)	37.4 (5.9)	**<0.001**
Resting heart rate (b·min^−1^)	78 (11)	74 (8)	0.27
Peak heart rate (b·min^−1^)	143 (17)	157 (11)	**0.02**
Respiratory Exchange Ratio	1.25 (0.1)	1.13 (0.1)	**0.001**
Power output at *V*O_2peak_ (W)	137 (27)	204 (38)	**<0.001**
Weekly physical activity (min)	446 (599)	836 (698)	**0.008**

*BMI, body mass index; VO_2peak_, peak oxygen consumption. All data are mean (SD) unless stated otherwise. P-values in bold represent a significant difference (P < 0.05)*.

### Experimental exercise responses

Mean power output (W) during exercise was greater in the higher-fit group [moderate-intensity continuous: mean = 81 W, (95% CI, 71–91); higher-intensity interval: mean = 143 W (95% CI, 133–152)] compared to the lower-fit group [moderate-intensity continuous: mean = 55 W (95% CI, 45–64); higher-intensity interval: mean = 96 W (95% CI, 86–105), *P* < 0.001]. Mean heart rate during higher-intensity interval exercise was greater in the higher-fit compared to the lower-fit group [mean heart rate 111 b·min^−1^ (95% CI, 105–117) vs. 103 b·min^−1^ (95% CI, 98–109), *P* = 0.05], but mean heart rate was similar between groups during moderate-intensity continuous exercise [98 b·min^−1^ (95% CI, 93–104) vs. 96 b·min^−1^ (95% CI, 91–101), *P* = 0.51)] and control [58 b·min^−1^ (95% CI, 52–63) vs. 63 b·min^−1^ (95% CI, 58–68), *P* = 0.18]. Mean RPE was 4 (95% CI, 3–4) during higher-intensity interval exercise compared to 3 (95% CI, 2–3, *P* < 0.001) in moderate-intensity continuous exercise, with no differences between groups (*P* = 0.50).

### Cytokine concentrations at baseline and in response to exercise

Plasma IL-6, IL-10, and TNF-α concentrations during each experimental visit (baseline and after different protocols) are reported in Tables 2, 3, and findings are summarized below.

**Table 2:** Plasma IL-6 and IL-10 concentrations before and after control, moderate- and higher-intensity exercise in higher and lower fit groups.

		**Time point (min)**				***P*****-value**			
		**Baseline**	**0 Post**	**20 Post**	**90 Post**	**Time**	**Protocol**	**Group**	**Interactions**
**Interleukin-6 (pg/ml)**									
Lower fit	Control	1.83 (1.37–2.29)	2.03 (1.61–2.46)^a^	1.93 (1.43–2.44)	2.15 (1.62–2.68)				Protocol*Time: 0.89 Protocol*Group: 0.06 Time*Group: 0.34 Protocol*Time*Group: 0.74
	Moderate-intensity	1.95 (1.46–2.44)	2.47 (1.91–3.03)^a^	2.08 (1.61–2.56)	2.59 (1.87–3.31)				
	Higher-intensity	2.15 (1.48–2.81)	2.27 (1.75–2.79)^a^	2.21 (1.53–2.90)	2.22 (1.72–2.72)	**0.02**	0.11	**0.02**	
Higher fit	Control	3.53 (2.24–4.82)	3.70 (2.38–5.01)^a^	3.48 (1.92–5.03)	3.53 (2.26–4.79)				
	Moderate-intensity	3.10 (1.93–4.28)	3.88 (2.55–5.21)^a^	3.58 (2.15–5.02)	3.26 (1.60–4.91)				
	Higher-intensity	3.56 (2.29–4.84)	4.14 (2.83–5.46)^a^	4.01 (2.50–5.51)	3.96 (2.51–5.41)				
**Interleukin-10 (pg/ml)**									
Lower fit	Control	1.59 (1.06–2.13)	1.71 (1.19–2.24)^a^	1.54 (1.06–2.02)	1.58 (1.11–2.04)				Protocol*Time: 0.83 Protocol*Group: < **0.01** Time*Group: 0.12 Protocol*Time*Group: 0.87
	Moderate-intensity	2.05 (1.11–3.00)^b^	2.19 (0.97–3.41)^a^^b^	1.97 (0.99–2.95)^b^	2.31 (0.85–3.78)^b^				
	Higher-intensity	1.59 (1.17–2.01)	1.70 (1.26–2.14)^a^	1.50 (1.04–1.97)	1.62 (1.21–2.02)	**<0.01**	**0.02**	0.45	
Higher fit	Control	2.51 (1.00–4.03)	2.50 (0.83–4.13)^a^	2.56 (0.57–4.54)	2.19 (0.72–3.65)				
	Moderate-intensity	2.32 (0.82–3.81)	2.71 (0.80–4.62)^a^	2.54 (0.73–4.35)	2.20 (0.71–3.70)				
	Higher-intensity	2.40 (0.76–4.03)	2.79 (0.97–4.62)^a^	2.75 (0.41–5.09)	2.38 (0.70–4.07)				

*Interleukin-6 (IL-6) and IL-10 responses to seated control, moderate-intensity continuous and higher-intensity intermittent exercise protocols. Data presented are from plasma samples taken before, immediately after, 20 and 90 min post-protocol. All data are mean (95% confidence interval)*.

a *Higher at 0 min post-protocol compared with baseline (P < 0.05)*.

b *Higher during moderate-intensity visit compared with higher-intensity and control visits in lower fit group only (P < 0.05). P-values in bold represent a significant difference (P < 0.05)*.

**Table 3:** Plasma TNF-α concentrations before and after control, moderate- and higher-intensity exercise in higher and lower fit groups.

**Tumor necrosis factor (pg/ml)**		**Time point (min)**			***P*****-value**			
		**Baseline**	**0 Post**	**90 Post**	**Time**	**Protocol**	**Group**	**Interactions**
Lower fit	Control	13.62 (0.67–26.57)	13.43 (0.91–25.95)	13.59 (−0.33–27.51)				Protocol*Time: 0.70 Protocol*Group: 0.61 Time*Group: 0.45 Protocol*Time*Group: 0.46
	Moderate-intensity	12.23 (1.64–22.82)	11.96 (1.39–22.52)	11.44 (1.73–21.15)				
	Higher-intensity	13.54 (1.01–26.07)	13.15 (0.95–25.34)	12.40 (1.08–23.71)	0.26	0.34	0.33	
Higher fit	Control	41.40 (3.70–79.14)	38.07 (4.16–71.98)	39.76 (1.55–77.96)				
	Moderate-intensity	43.38 (4.42–82.33)	45.21 (3.62–86.81)	32.78 (2.94–62.62)				
	Higher-intensity	43.21 (0.55–85.87)	46.34 (1.84–90.85)	43.15 (0.47–85.82)				

*Tumor Necrosis Factor (TNF-α) responses to seated control, moderate-intensity continuous and higher-intensity intermittent exercise protocols. Data presented are from plasma samples taken before, immediately post- and 90 min post-protocol. All data are mean (95% confidence interval)*.

#### Cytokine concentrations at baseline

Mean plasma IL-6 concentration at baseline across all experimental visits was 1.4 pg/ml higher in the higher-fit group compared to the lower-fit group (95% CI, 0.2–2.6, *P* = 0.02). There was a trend toward higher mean TNF-α concentrations in the higher-fit group compared to the lower-fit group (*P* = 0.16), and no *group* differences in mean IL-10 concentrations at baseline. There was no difference in cytokine concentrations at baseline between visits (*P* > 0.05).

#### Cytokine concentrations in response to exercise

There was a time effect where mean IL-6 concentration in participants from both fitness groups increased by 0.4 pg/ml (95% CI, 0.1–0.7, *P* = 0.006) immediately post-protocol before returning to baseline values. Similarly, mean IL-10 concentration in participants from both fitness groups increased by 0.2 pg/ml (95% CI, 0.0–0.4, *P* = 0.002) immediately post-protocol before returning to baseline values. However, there were no group^*^time or protocol^*^time interactions for IL-6 or IL-10, indicating that the IL-6 and IL-10 responses were consistent between fitness groups and were not different between exercise and non-exercise protocols (Table 2). To account for the small baseline differences in IL-6 concentrations, analyses were repeated using delta (change from baseline) responses; however, this analysis did not change the findings (group^*^time^*^protocol interaction, *P* = 0.861). No differences in TNF-α concentrations were found over time, between groups or between protocols (Table 3).

### Relationships between physical activity, fitness, and cytokine concentrations

Despite a significant difference in IL-6 concentration at baseline between lower and higher-fit groups, there was no correlation between IL-6 at baseline and *V*O_2peak_ (*r* = 0.29, *P* = 0.12) in all participants. Higher IL-10 and TNF-α concentrations at baseline were associated with increased *V*O_2peak_ (IL-10: *r* = 0.43, *P* = 0.02; TNF-α: *r* = 0.38, *P* = 0.04). No correlations were found between self-reported physical activity levels and IL-6, IL-10, or TNF-α concentrations at baseline. In addition, there were no correlations between self-reported physical activity levels or *V*O_2peak_, and changes in cytokine concentrations after moderate-intensity continuous exercise or higher-intensity interval exercise.

## Discussion

This is the first study to compare the effects of lower and higher cardiorespiratory fitness on the circulating cytokine (IL-6, IL-10, and TNF-α) responses to short-term exercise in healthy older individuals. Our findings suggest that a single bout of moderate-intensity continuous or higher-intensity interval cycling exercise does not elicit a measurable cytokine response in healthy older adults, and these responses were similar in older adults with higher or lower levels of cardiorespiratory fitness.

### Effect of cardiorespiratory fitness on basal cytokine levels

We observed significantly higher mean IL-6 at baseline in the higher-fit group (3.4 pg/ml) compared to the lower-fit group (2.0 pg/ml, *P* = 0.02), with no group differences in IL-10 or TNF-α concentrations. Contrary to our findings, higher levels of cardiorespiratory fitness have previously been associated with lower circulating cytokine levels in young (Kullo et al., 2007; Gaeini et al., 2009; Lin et al., 2010), older (Valentine et al., 2009) and diseased populations, such as those with cancer (Jones et al., 2008). Conversely, recreationally active vs. endurance trained younger individuals show similar IL-6 or TNF-α concentrations at baseline, despite large differences in cardiorespiratory fitness (Scott et al., 2013; Landers-Ramos et al., 2014).

In an attempt to explain the higher IL-6 concentration observed at baseline in the higher-fit compared to the lower-fit group in this study, we considered whether the higher physical activity levels reported by the higher-fit group in the preceding 7 days before testing may have impacted on the observed cytokine concentrations. The circulating IL-6 response to exercise is transient, typically reaching a peak upon the cessation of exercise and returning to basal levels within 24 h (Leggate et al., 2010; Mendham et al., 2011; Perandini et al., 2015). The cytokine concentrations observed in the current study are unlikely to have been altered by a prior exercise bout, as the participants were asked to refrain from exercise or strenuous physical activity for at least 24 h prior to each experimental visit. While high physical activity levels such as those seen in the higher-fit group are typically considered to be beneficial for immune function (Estrela et al., 2017; Minuzzi et al., 2017), a U-shaped relationship between physical activity levels and infection (and subsequent inflammation) risk has also previously been proposed (Walsh et al., 2011; Gleeson and Walsh, 2012; Turner, 2016). Specifically, circulating inflammatory markers, such as IL-6, may be elevated in those undertaking heavy training loads. Despite this, we found no relationship between basal IL-6, IL-10, or TNF-α concentrations and self-reported physical activity levels in the preceding 7 days, suggesting that higher physical activity levels did not contribute to high circulating cytokines at baseline in our higher-fit group. After careful consideration, the cause of the higher basal IL-6 concentrations observed in the higher-fit group compared to the lower-fit group in this study is unclear. It is also unclear whether the higher basal circulating concentrations of IL-6 observed in the higher-fit group are indicative of a pro- or anti-inflammatory response. Further research of this complex cytokine incorporating comprehensive measurements of immune function is warranted to assess the effect of cardiorespiratory fitness and physical activity on basal inflammation in older adults.

### Cytokine responses to exercise in older adults

Unlike many previous studies assessing cytokine responses to exercise, the experimental design of this study included a non-exercise control condition. Interestingly, we observed an increase in IL-6 and IL-10 concentrations immediately after all experimental protocols, with no difference in the cytokine response between the exercise and non-exercise protocols. These findings should be considered when interpreting previous studies, and highlight the importance of including a non-exercise protocol in the experimental design of future studies to prevent the reporting of false positive cytokine responses to exercise.

The exercise protocol intensity and duration used in this study is consistent with the current exercise prescription guidelines for health in older adults. Despite this, we observed no exercise-induced cytokine responses to exercise. These findings support the suggestion that acute cytokine responses to exercise may be blunted in older adults (Hamada et al., 2005; Reihmane et al., 2016) compared to previous observations in younger adults (Reihmane et al., 2013). Further studies comparing acute cytokine responses to short-term exercise in older and younger participant groups would help further clarify the effect of age on the inflammatory response to exercise.

### Effect of cardiorespiratory fitness on cytokine responses to exercise in older adults

There was no difference in the acute cytokine responses to a bout of exercise between older adults with lower and higher levels of cardiorespiratory fitness. These, findings concur with previous cross-sectional observations in young adults (Scott et al., 2013; Landers-Ramos et al., 2014) and middle-aged adults (Minuzzi et al., 2017). Studies assessing acute cytokine responses to exercise in younger adults have only included individuals with above-average levels of cardiorespiratory fitness, relative to population normative values (Scott et al., 2013; Landers-Ramos et al., 2014), which did not rule out the possibility that low levels of fitness might impact the cytokine response to exercise. The current study is the first to compare acute cytokine responses to exercise in participants with above average (37.4 ± 5.9 mL.kg^−1^.min^−1^) and below average (22.6 ± 2.8 mL.kg^−1^.min^−1^) cardiorespiratory fitness levels, relative to age-specific normative data (Stensvold et al., 2017). Our findings indicate that the blunted cytokine response to exercise previously reported among older adults (Hamada et al., 2005; Reihmane et al., 2016) is not likely to be due to low fitness levels.

In addition to having higher levels of cardiorespiratory fitness, the higher-fit group in this study were more physically active than the lower-fit group. However, we observed no relationships between self-reported physical activity levels and IL-6, IL-10, or TNF-α changes in response to exercise. These findings support previous observations in older individuals (Estrela et al., 2017). Estrela et al. (2017) found no difference in the IL-6 or TNF-α response to two successive bouts of maximal exercise between marathon runners with higher (~480 min/week) and lower training volumes (~240 min/week) (Estrela et al., 2017). Taken together, the findings of the current study and those reported previously suggest that neither cardiorespiratory fitness nor physical activity levels alter the acute cytokine responses to exercise in healthy older adults, and these findings appear to be consistent following both maximal and submaximal exercise.

Cytokine responses to exercise in all populations are known to be positively associated with both exercise intensity and duration (Fischer, 2006). Therefore, we adopted work- and time-matched exercise protocols to investigate the effect of exercise intensity on cytokine responses to exercise. We observed no differences in cytokine responses to moderate-intensity continuous exercise when compared to higher-intensity interval exercise of the same duration and total external workload. These findings contrast with previous observations of augmented IL-6 release in younger adults after interval exercise when compared to work- and time-matched continuous exercise (Leggate et al., 2010), albeit after longer duration exercise (58 min compared to 24 min in the current study). Further, we previously found that the IL-6 response was lower after interval exercise compared to continuous exercise (Windsor et al., 2017), although that study only included individuals with relatively low levels of cardiorespiratory fitness. Exercise duration has been reported to be the single most important factor determining the amplitude of the IL-6 response to exercise, accounting for more than 50% of the variation in plasma IL-6 (Fischer, 2006). Thus the contrasting findings between younger adults (Leggate et al., 2010) and older adults in the current study may be due to differences in the duration (and volume) of exercise. Taken together, the effect of exercise intensity on cytokine responses to exercise in older adults is still unclear, and the effect of exercise intensity in older adults after longer duration exercise is still to be determined.

When interpreting the findings of this study, it should be considered that we did not control for exercise induced changes in plasma volume. However, exercise induced fluid shifts in response to <30 min of submaximal exercise are typically small (Zouhal et al., 2001), and previous studies using a similar duration exercise protocol have found no changes in haematocrit or hemoglobin concentrations (Landers-Ramos et al., 2014). In addition, the non-exercise control visit in this study allowed us to highlight any non-exercise related variance in plasma cytokine concentrations such as those caused by postural-induced changes in plasma volume.

## Conclusion

Shorts bouts of moderate-intensity continuous and higher-intensity interval cycling exercise did not influence circulating cytokine concentrations in healthy older adults, and this response was similar in older adults with higher or lower levels of cardiorespiratory fitness. The lack of an exercise-induced cytokine response in either group may suggest that the acute inflammatory response to short-term exercise is blunted in older adults, irrespective of cardiorespiratory fitness level. Further studies comparing cytokine responses to short-term exercise in older and younger adults would help clarify the effect of age on the inflammatory response to exercise.

## Author contributions

MW, TB, MP, JG, FR, and CA were responsible for the study conception and design. MW, TB, MP, LM, FR, and CA were responsible for the acquisition of data. MW, TB, LM, FR, and CA were responsible for the analysis and interpretation of data. MW, TB, and CA were responsible for drafting the manuscript. MW, TB, MP, LM, JG, FR, and CA were responsible for critical revision of the manuscript. All authors approved the final version of the manuscript for publication.

### Conflict of interest statement

The authors declare that the research was conducted in the absence of any commercial or financial relationships that could be construed as a potential conflict of interest.
